# The precision of gingival recession measurements is increased by an automated curvature analysis method

**DOI:** 10.1186/s12903-021-01858-9

**Published:** 2021-10-07

**Authors:** Marko Kuralt, Rok Gašperšič, Aleš Fidler

**Affiliations:** 1 Department of Restorative Dentistry and Endodontics, University Medical Centre Ljubljana, Hrvatski trg 6, 1000 Ljubljana, Slovenia; 2 Faculty of Medicine, University of Ljubljana, Ljubljana, Slovenia; 3 Department of Oral Medicine and Periodontology, University Medical Centre Ljubljana, Ljubljana, Slovenia; 4 Department of Oral Medicine and Periodontology, Faculty of Medicine, University of Ljubljana, Ljubljana, Slovenia; 5 Department of Endodontics and Operative Dentistry, Faculty of Medicine, University of Ljubljana, Ljubljana, Slovenia

**Keywords:** Gingival recession, Computer-assisted image processing, Dental models, Cementoenamel junction, Observer variation, Computer-assisted image interpretation reproducibility of results

## Abstract

**Background:**

The extent of gingival recession represents one of the most important measures determining outcome of periodontal plastic surgery. The accurate measurements are, thus, critical for optimal treatment planning and outcome evaluation. Present study aimed to introduce automated curvature-based digital gingival recession measurements, evaluate the agreement and reliability of manual measurements, and identify sources of manual variability.

**Methods:**

Measurement of gingival recessions was performed manually by three examiners and automatically using curvature analysis on representative cross-sections (n = 60). Cemento-enamel junction (CEJ) and gingival margin (GM) measurement points selection was the only variable. Agreement and reliability of measurements were analysed using intra- and inter-examiner correlations and Bland–Altman plots. Measurement point selection variability was evaluated with manual point distance deviation from an automatic point. The effect of curvature on manual point selection was evaluated with scatter plots.

**Results:**

Bland–Altman plots revealed a high variability of examiner’s recession measurements indicated by high 95% limits of agreement range of approximately 1 mm and several outliers beyond the limits of agreement. CEJ point selection was the main source of examiner’s variability due to smaller curvature values than GM, i.e., median values of − 0.98 mm^− 1^ and − 4.39 mm^− 1^, respectively, indicating straighter profile for CEJ point. Scatter plots revealed inverse relationship between curvature and examiner deviation for CEJ point, indicating a threshold curvature value around 1 mm^− 1^.

**Conclusions:**

Automated curvature-based approach increases the precision of recession measurements by reproducible measurement point selection. Proposed approach allows evaluation of teeth with indistinguishable CEJ that could be not be included in the previous studies.

**Supplementary Information:**

The online version contains supplementary material available at 10.1186/s12903-021-01858-9.

## Background

The extent of gingival recession, i.e., recession depth, is evaluated before and after treatment and represents one of the most important measures determining treatment outcome [1]. Recession depth is a measure used to quantify treatment success in terms of percentage of root coverage and defects with complete root coverage, enabling comparison between different treatment techniques [1, 2]. Therefore, the accuracy of recession depth measurements is critical for diagnosis, optimal treatment planning, and outcome evaluation.

The standard method for gingival recession evaluation is an assessment with the periodontal probe, allowing precise evaluation at a clinically acceptable level [3, 4]. However, the following limitations should be considered, i.e., variations in position and angulation of the periodontal probe and rounding errors [5–7]. Rounding errors were eliminated with utilization of digital callipers with resolution of 0.01 mm either with pair of dividers [8] or endodontic spreader [9]. While, 3D digital measuring method [10, 11] eliminated both limitations. In brief, the measurement site and direction were defined by selecting a cross-section aligned with the tooth’s long axis, eliminating the variations in position and angulation of the periodontal probe. At the same time, utilisation of a digital ruler allowing for measurements to the nearest 0.01 mm increased the measurement’s accuracy and eliminated rounding of measurements to the nearest mm as with a periodontal probe.

By definition, recession depth is defined as a distance between anatomical landmark, i.e., the cemento-enamel junction (CEJ), and gingival margin (GM) [12]. Thus, the accuracy of recession depth measurements strongly depends on the measurement point selection. The precise determination of the CEJ was already outlined as a significant limitation, reducing the reliability and reproducibility of recession depth measurements [13, 14]. Furthermore, precise localisation of CEJ is hampered by poor visibility, carious or non‐carious cervical lesions or cervical restorations [15, 16]. Pini-Prato et al. [17] showed that almost 40% of teeth with gingival recession exhibit unidentifiable CEJ, rendering repeated measurements using the CEJ nearly impossible and prone to errors during follow-up.

By definition, from medical image analysis, landmarks are well-defined points that can be identified based on distinguishable shape features and can be placed manually by examiners or detected automatically [18, 19]. With recent advancements, automated image analysis based on geometric properties is gaining increased importance in dentistry [20–24]. One of the widely used geometric properties is curvature [25, 26], a mathematical measure describing a deviation of a curve from being a straight line. In dentistry, curvature analysis was already applied for facial profile landmarking in orthodontics [27] and teeth dimension measurements [28], exhibiting reliable and reproducible evaluation. Up to now, the curvature analysis has not been used in the evaluation of gingival recessions, despite wide variety of utilized digital methods [29].

The present study aimed to (1) introduce automated digital measurements of recession depth by using curvature analysis, (2) evaluate the agreement and reliability of manual digital measurements with examiners with different level of experience, (3) identify the sources of examiner’s variability by using curvature analysis, and (4) evaluate the agreement of automated digital and clinical measurements.

## Methods

### Sample

An existing dataset of digital dental models of patients presenting with gingival recessions referred to the Department of Oral Medicine and Periodontology, University Medical Centre Ljubljana, Slovenia, was used to compare digital measurements of gingival recessions. The digital dental models were acquired by intraoral scanning (CEREC Omnicam AC, Dentsply Sirona; software version: SW 4.5.2) when the first author, i.e., experienced operator, was present at the Department in the year 2019. Thus, the dataset is representing a convenience sample. The National Medical Ethics Committee approved the study (Protocol No. 0120-595/2018/4), and the study was conducted in accordance with the Helsinki Declaration as revised in 2013.

The complete dataset was screened for study eligibility by the first author prior to study entry. Digital dental models were eligible for inclusion when gingival recession defect was present at any tooth either in the upper or lower arch and only when the affected teeth presented with identifiable natural CEJ, i.e., Class A- and A + in the Classification of root surface concavities by Pini-Prato et al. [17]. In cases where multiple digital dental models from the same patient were available, e.g., follow-up models after root coverage, only the baseline model was used. In patients presented with multiple gingival recessions, each gingival recession defect was referred to as an individual unit.

The study sample consisted of digital dental models obtained from ten patients, resulting in 52 teeth with gingival recession with identifiable natural CEJ (Additional file 1). Due to the inclusion of molars with the gingival recession at both roots at the buccal side, 60 measuring sites were collected. The distribution of measuring sites per tooth group were: 10 incisors (7 maxillary and 3 mandibular), 10 canines (9 maxillary and 1 lower), 20 premolars (18 maxillary and 2 lowers), and 20 molars (all maxillary).

### Comparison of digital measurements of recession depth

Included digital dental models were then imported into the 3D data measurement analysis software (GOM Inspect 2017; GOM GmbH). A local coordinate system was created for each tooth of interest, with a long axis of the tooth matching the *z*-axis and mesio-distal direction matching the *x*-axis. Digital dental models were refined with “Refine mesh” tool (with “Minimal edge length” setting set at 0.01 mm) to enhance the digital model quality and resolution. A representative bucco-oral cross-section was then manually selected at the central buccal site in the direction of the tooth’s long axis. In molars, a bucco-oral cross-section was created for each root if the gingival recession was present at the corresponding root.

Measurements of gingival recessions were performed independently by manual and automatic approach (Additional file 2). In the cross-section images, two measurement points, representing CEJ and GM, were determined manually and automatically, followed by an automated distance measurement.

**Fig. 1:**
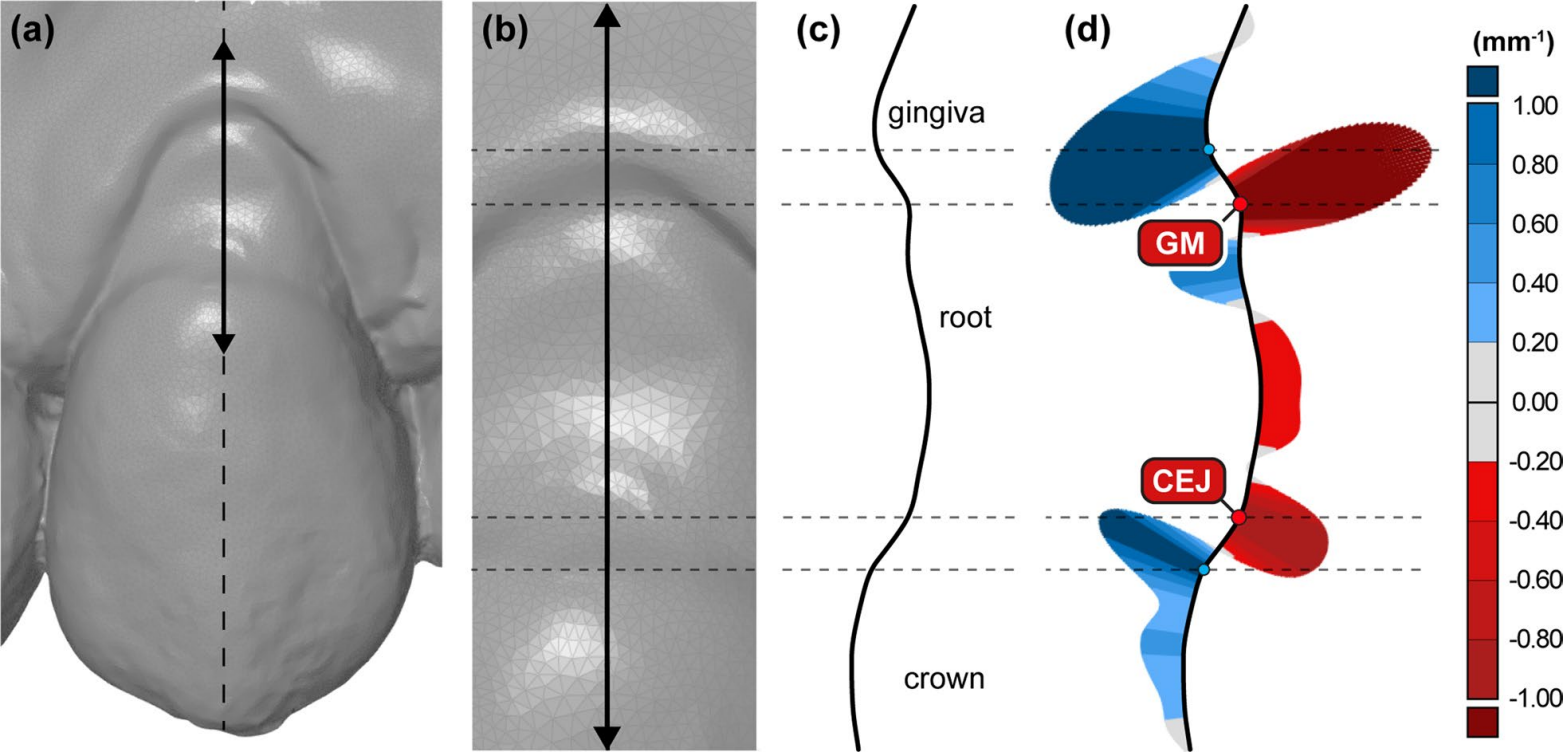
Automated measurement point selection by using curvature analysis on a representative cross-section. Digital model of maxillary left canine with a representative cross-section at the central buccal site in the direction of the long axis of the tooth (full black line) (**a**) and a corresponding close-up view (**b**). A side view of a cross-section (**c**) also depicting the view for manual measurement point selection. A “curvature comb”, i.e., a graph that displays the curvature values at a specific point on an actual cross-section, is shown at (**d**). Two cross-section segments of interest are marked with dashed grey lines, defined with two local extreme points with positive and negative curvature, i.e., maximum convexity (blue) and concavity (red), respectively. Both cemento-enamel junction (CEJ) and gingival margin (GM) points are defined as a local extreme point with maximum concavity (red) within the cross-section segments mentioned above

For the manual method, three examiners with various level of digital image analysis experience performed the measurement point selection on the cross-sections (Fig. 1a–c). Examiner No. 1, general dental practitioner with 9 years of clinical experience, was without previous experience in digital image analysis. Examiner No. 2, endodontist, was experienced in digital analysis with over 20 years of experience. Examiner No. 3, periodontist, was without previous experience in digital image analysis, but was experienced in clinical diagnostics, i.e., a standard calibrated examiner for clinical periodontal measurements. The process of measurement point selection was repeated one week later by all three examiners. The order of cross-sections was randomised for each examiner, reducing the possible bias in the repeated measurements.

For the automatic method, the shape of each cross-section was mathematically described by the curvature. Curvature was computed by the software’s “Compute Curve Curvature” tool describing a cross-section curve with a mathematical function, i.e., spline function. From this spline curve, curvature values were determined for each point at a distance of 0.01 mm. Obtained curvature values were then visualised with colour-coded “curvature combs” [30], i.e., graphs that display the amount of curvature at a specific point on the cross-section (Fig. 1d).

Based on the obtained curvature values, two cross-section segments of interest were recognised on all cross-sections, i.e., CEJ and GM. CEJ segment is represented by the transition from convex to concave shape in corono-apical direction. In contrast, the GM segment is represented by the transition from convex to concave shape in the opposite direction, i.e., apico-coronal. For precise measurement point selection, local extreme curvature values in the concave part of both segments mentioned above were determined as CEJ and GM points (Fig. 1d). GM point was determined based on the established gingival margin definition [11, 31]. CEJ point decision was based on existing literature [28, 32–34]. After the determination of points of interest, points are then selected automatically.

After either manual or automatic measurement point selection, a gingival recession was measured automatically as a distance between the measurement points.

### Evaluation of manual measurement point selection variability

Measurement point selection variability was measured as a distance of manual measurement point in reference to automated curvature-based point position on a cross-section. The distance was obtained with the software’s “Arc length” tool. It was performed for both the CEJ and GM measurement points. Results were presented using box-plots with positive values representing coronal deviation and negative values apical deviation of manual measurement points.

Furthermore, for CEJ measurement point, the effect of curvature on manual point selection deviation was evaluated by creating curvature vs. examiner deviation scatter plots.

### Comparison of digital and clinical recession depth measurements

Obtained automated and manual digital measurements were compared to clinical measurements of recession depth. Clinical measurements were collected retrospectively from patient’s records and were performed by Examiner No. 3.

### Statistical analysis

Statistical analysis was carried out using a statistical software program (IBM SPSS Statistics version 25; IBM Corp). Descriptive statistics, including mean, standard deviations, median, minimum, and maximum values, were obtained.

For evaluation of the examiners’ bias, i.e., reliability or inter-examiner variability, and ability of the examiners to repeat multiple measurements, i.e., reproducibility or intra-examiner variability, the intraclass correlation coefficient (ICC) and confidence intervals were calculated.

Agreement of the digital measurements between examiners was analysed using Bland–Altman plots using the curvature-based approach as a reference. The mean differences between tested groups were assessed using the one-sample t-test to determine if mean differences were statistically different from zero.

Agreement of the clinical and digital approach was also analysed using Bland–Altman plot using the clinical approach as a reference and the mean differences between tested groups assessed using the one-sample t-test.

## Results

### Recession depth measurements

Median recession depth obtained with an automated curvature-based approach for a study sample (n = 60) was 1.90 mm (25th–75th percentile: 1.25–2.62 mm), representing a sample of predominately shallow gingival defects.

### Agreement and reliability of digital recession depth measurements

Based on the inter- and intra-examiner reliability with high lower boundary values of the 95% confidence intervals, i.e., 0.93 and 0.94, respectively, the manual approach can be considered excellent regarding precision.

**Fig. 2:**
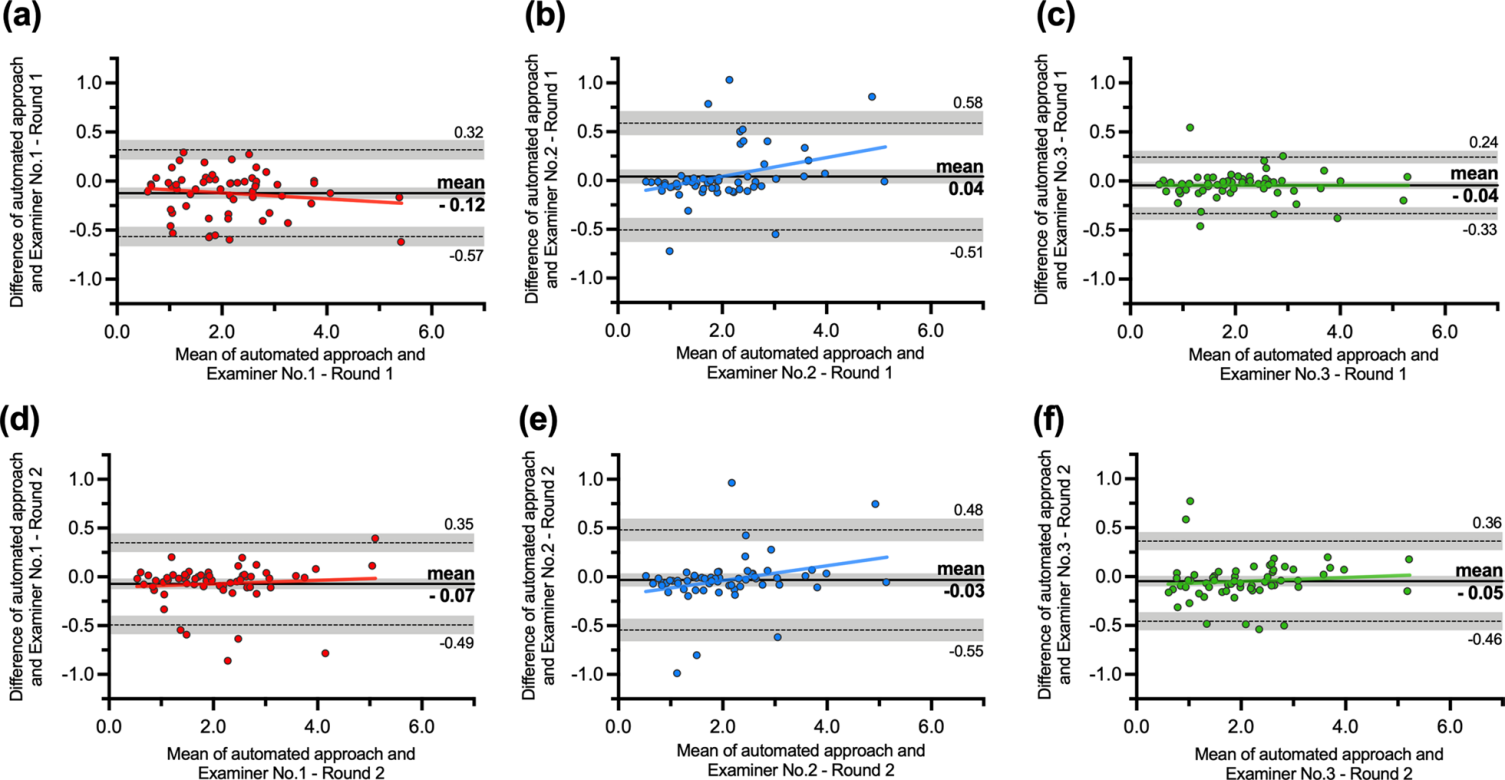
Bland–Altman plots showing a comparison of digital measurements of gingival recession between Examiner No. 1 (red dots), No. 2 (blue dots), and No. 3 (green dots) in the first (**a**–**c**) and second round of measurements (**d**–**f**) in reference to automated curvature-based approach. The *x*-axis indicates the mean measurement of the gingival recession between compared approaches. The *y*-axis indicates the difference between compared approaches. A black line with the surrounding grey area indicates mean bias and 95% confidence interval. A dashed black line with a surrounding grey area indicates either upper or lower 95% limits of agreement and corresponding 95% confidence interval

The mean differences (and 95% confidence interval) between the manual and automated approach on Bland–Altman plots were around zero, i.e., − 0.12 mm (− 0.18 – − 0.07), 0.04 mm (− 0.03–0.11), and − 0.04 mm (− 0.08 – − 0.01) for Examiner No. 1, 2, and 3 in Round 1, respectively, and − 0.07 mm (− 0.13 – − 0.02), − 0.03 mm (− 0.10–0.04), and − 0.05 mm (− 0.10–0.01) for Examiner No. 1, 2, and 3 in Round 2, respectively (Fig. 2). A statistically significant mean difference after adjustment for multiple comparisons was found only for Examiner No.1 in the first round of measurements compared to the automated measurements (*p* < 0.001) (Fig. 2a). The high variability of recession depth measurements was found for all three examiners in both rounds, indicated by high 95% limits of agreement range of approximately 1 mm and outliers beyond the limits of agreement (Fig. 2).

### Manual measurement point selection deviation

**Fig. 3:**
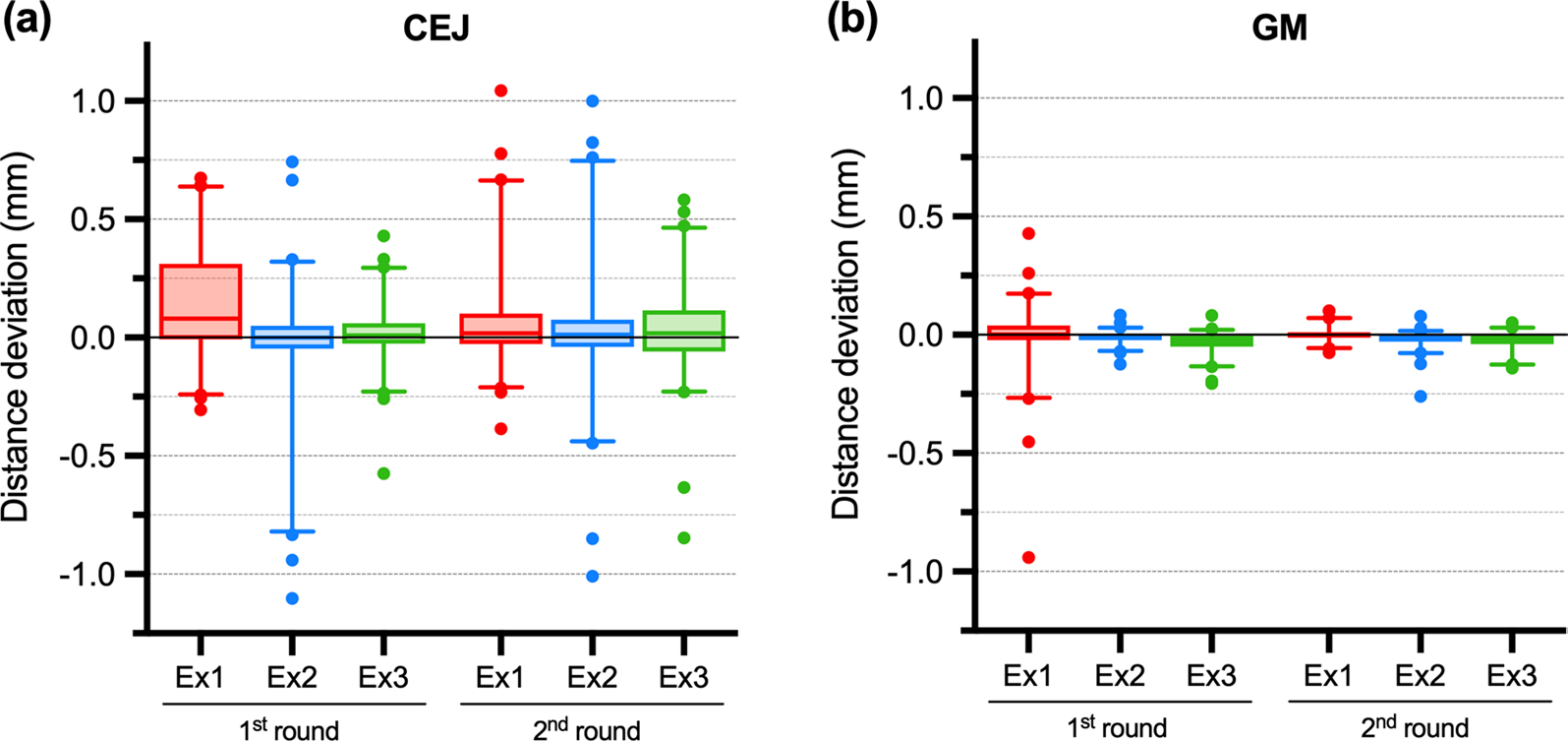
Manual measurement point selection variability depicted with box-plots with measurement point distance deviation of cemento-enamel junction (CEJ) (**a**) and gingival margin (GM) point (**b**) for each examiner and a round of measurement in reference to automated curvature-based point position on a cross-section. Box represent 25th and 75th percentile, while whisker represents 5th and 95th percentile and dots represent outliers

A small median difference was found for both CEJ and GM points, except for Examiner No. 1’s first-round (Fig. 3). High variability was found for CEJ compared to the GM point, indicated by a high interquartile range and several outliers (Fig. 3a).

**Fig. 4:**
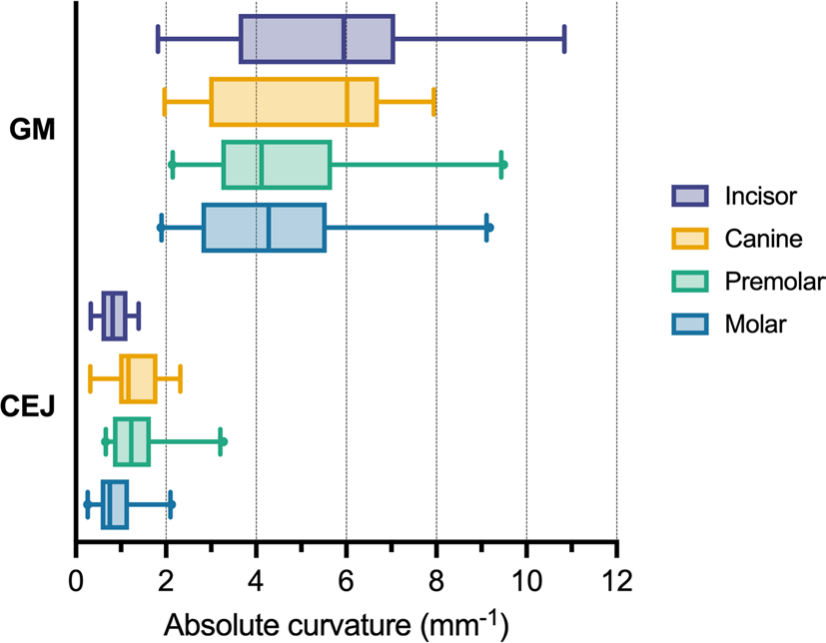
Profile shape description of cemento-enamel junction (CEJ) and gingival margin (GM) points using box-plots split by tooth group. Box represent the 25th and 75th percentile, while whiskers represent the 5th and 95th percentile, and dots represent outliers

**Fig. 5:**
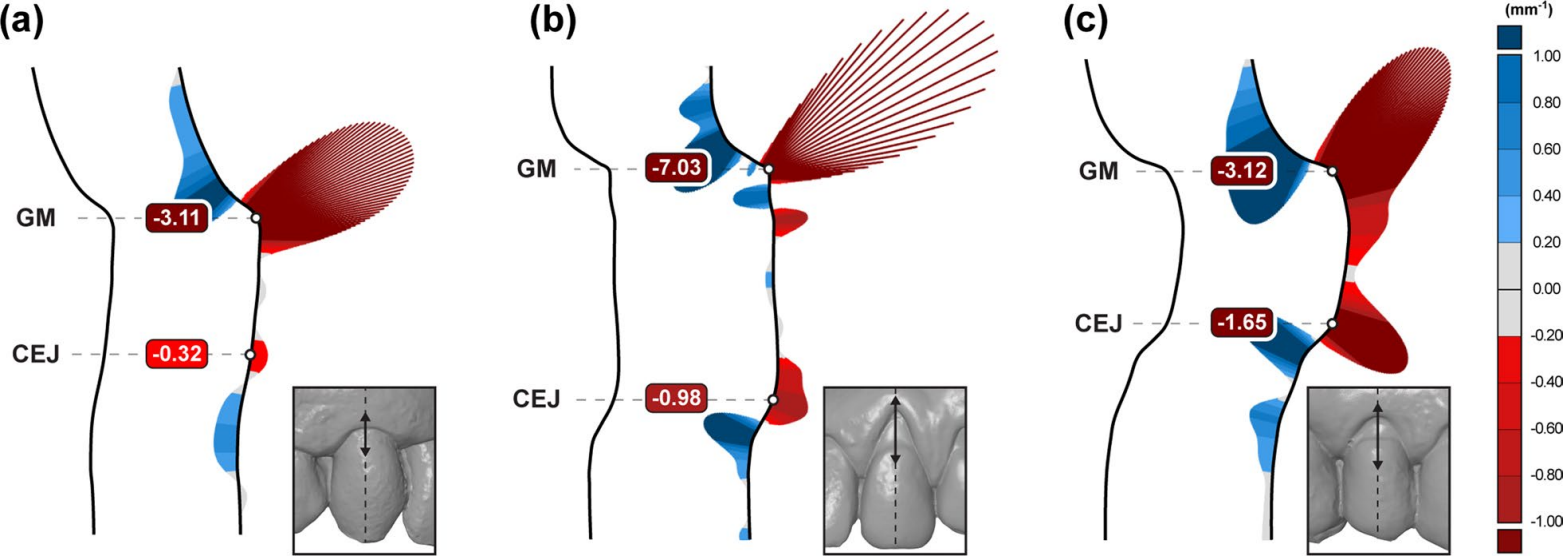
Cross-section shapes of representative gingival margin (GM) and cemento-enamel junction (CEJ) profiles with “curvature combs” graphs of three maxillary teeth, i.e., right canine (**a**), left canine (**b**), and right first premolar (**c**). Visual inspection of cross-section profiles and “curvature comb” graphs depicts GM point as more distinct and easier to select than CEJ point. Visually perceived shape differences at CEJ points compared to GM points are larger despite the smaller differences in curvature values, i.e., − 0.32 mm^− 1^ vs. − 0.98 mm^− 1^ for CEJ points and − 3.11 mm^− 1^ vs. − 7.03 mm^− 1^ for GM points (**a** and **b**, respectively). It results from the mathematical definition of curvature as a ratio, i.e., an inverse radius (1/r), and human perception, which more easily distinguish between the straight line and a circle than between two circles with a small difference in radius. Interestingly, similar GM curvature values in **a** and **c** produce different shapes of curvature comb graphs (figure **c** “curvature comb” in coronal direction), which can be explained as an effect of root surface defect present in **c**

The overall shape description of CEJ and GM profiles using curvature showed that CEJ points exhibit lower curvature values than GM points, with median (5th–95th percentile) of 0.99 mm^− 1^ (0.32–2.30) and 4.39 mm^− 1^ (1.98–9.14) for CEJ and GM, respectively. Values and box-plots also revealed that CEJ points exhibit a smaller range than GM points (Fig. 4), despite visually more considerable shape differences between CEJ profiles, as depicted by visual inspection of cross-sections (Fig. 5).

**Fig. 6:**
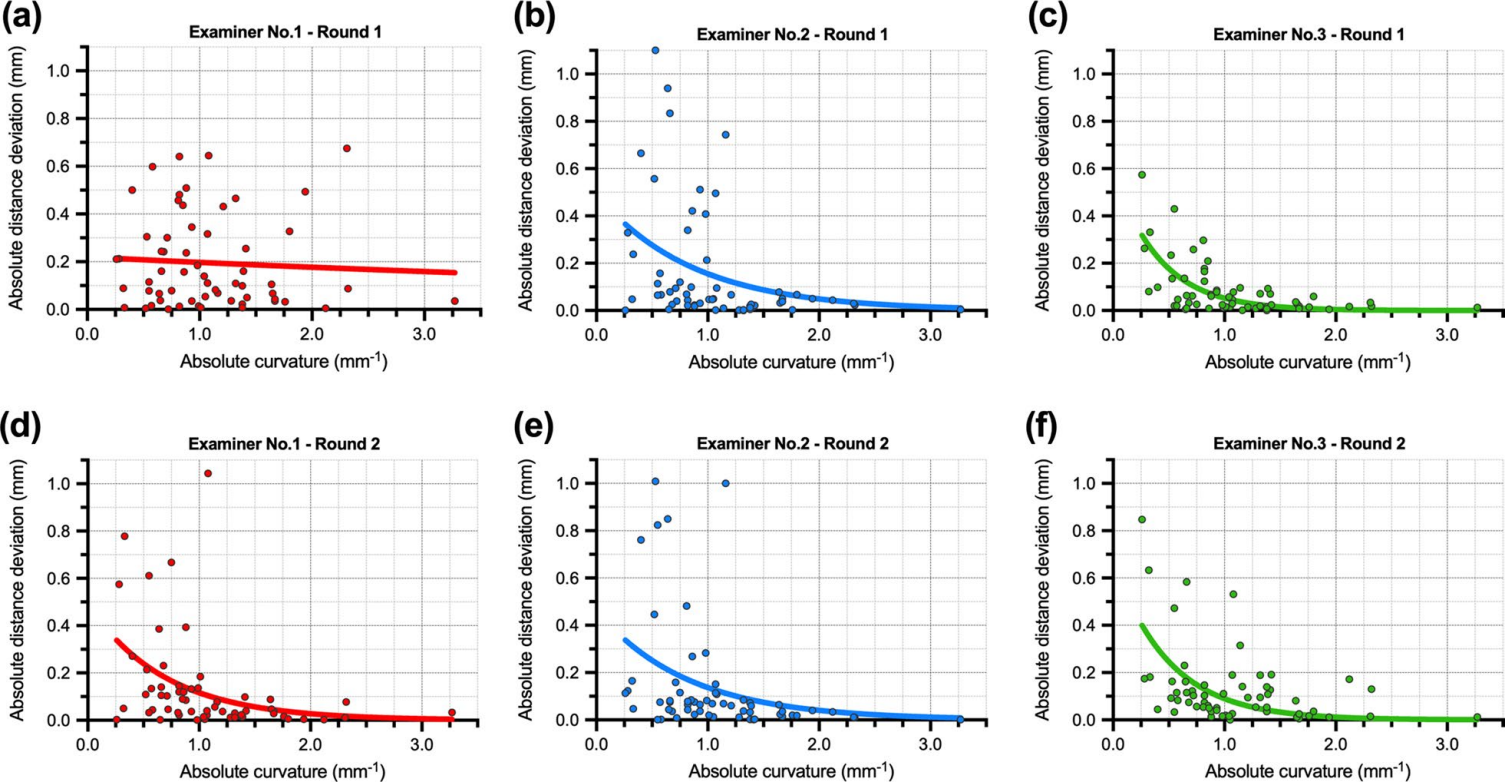
Scatter plots displaying correlation of curvature and manual CEJ points distance deviation, i.e., the distance between manual and automated curvature-based CEJ reference point position. The upper row shows the first round of measurement (**a**–**c**), and the lower row shows the second round of measurements (**d**–**f**). The same colour legend is used as in Figs. 2 and 3 and i.e., Examiner No. 1—red, No. 2—blue, and No. 3—green

The scatter plots revealed inverse relationship between curvature and examiner deviation distance for CEJ point, indicating a threshold curvature value around 1 mm^− 1^ (Fig. 6), with the exception of Examiner No. 1’s first round of measurements (Fig. 6a). Below this threshold value, the variability of deviation increase.

### Agreement of digital and clinical recession depth measurements

The mean differences (and 95% confidence interval) between the clinical and manual digital approach on Bland–Altman plots were 0.40 mm (0.20–0.59), 0.56 mm (0.39–0.73), and 0.48 mm (0.30–0.66) for Examiner No. 1, 2, and 3 in Round 1, respectively, and 0.45 mm (0.27–0.62), 0.49 mm (0.32–0.66), and 0.47 mm (0.29–0.66) for Examiner No. 1, 2, and 3 in Round 2, respectively (Additional file 3: Fig. S1). The mean difference (and 95% confidence interval) between the clinical and automated digital approach was 0.52 mm (0.34–0.70). A statistically significant mean difference after adjustment for multiple comparisons was found for all comparisons. The high variability of recession depth measurements was found for the clinical approach, indicated by high 95% limits of agreement range of approximately 2.7 mm (Additional file 3: Fig. S1).

## Discussion

Accurate evaluation of gingival recession dimensions is an essential part of the diagnosis, treatment planning, and outcome evaluation. In the present study, curvature analysis was used to accurately automate the measurement point selection required for recession depth measurement and analyse the examiners’ measurement error. The main source of variability for examiners was CEJ measurement point selection. The automated measurements of recession depth using curvature analysis reduce human variability and increase the precision of measurements.

In the present study, a sample of predominately shallow gingival defects was used to compare approaches due to the inclusion of teeth with identifiable natural CEJ only. Shallow gingival defects presenting with small recession depths are hard to evaluate with a periodontal probe due to mentioned limitations of measurement accuracy [5–7]. However, as already outlined by Zuhr et al. [10], increased resolution of digital measurements to the nearest 0.01 mm enables evaluation of shallow gingival defects as well. Additionally, the importance of measurement accuracy was also emphasised with a superb illustration of the effect of rounding digital measurements on two important parameters for evaluating the success of different treatment techniques, i.e., percentage of root coverage [35] and percentage of defects with complete root coverage [10, 35].

Manual digital measurements of gingival recession exhibit high variability depicted by Bland–Altman plots. Errors are an inherent part of manual measurements and are unavoidable with human involvement [36]. Therefore, for objective comparison of variability between examiners and the unknown true value of recession depth, an automated curvature-based approach was used as a reference method due to automaticity enabling perfect reproducibility of repeated measurements. Despite excellent ICC values and non-significant mean differences between the approaches, Bland–Altman analysis facilitated the comparison of digital approaches in each individual measurement and allowed for the detailed investigation of the performance of each approach in the sample, revealing a relatively high variability of recession depth measurements with a 95% limit of agreements range of approximately 1 mm. Despite inexperience in digital analysis, Examiner No. 3, being experienced in clinical diagnostics, exhibited the smallest range of 95% limits of agreement, i.e., 0.57 mm; however, no significant differences can be observed between examiners. Obtained variability range is much smaller than evaluation with a periodontal probe, i.e., around 2.5 mm, and similar to evaluation with a digital manual approach, i.e., around 1 mm, supported by our results (Additional file 2: Fig. S1) and also found in other studies [3, 4]. In contrast to previous digital studies, the only variable part in the present study was measurement point selection; thus, obtained variability can be attributed solely to the measurement points selection. The digital manual approach utilised in the previous studies [3, 4] measured gingival recessions on the digital models and not on cross-sections, including measuring direction and angle variability, into the comparison. The implementation of the proposed approach is straightforward. It requires only a single 3D data measurement analysis software that is free for non-commercial use.

Importantly, the main source of variability is the CEJ measurement point. Both the manual and the automated approach used the shape of the cross-section for the measurement point selection. The main difference between the approaches was the examiners’ subjective bias. Our results revealed that the main variability could be attributed to the position of the CEJ measurement point (Fig. 3a), despite inclusion criteria with visible and identifiable CEJ. Curvature analysis revealed a straighter CEJ profile compared to the GM profile, outlined with smaller absolute curvature values (Fig. 4), resulting in a less distinct shape feature for CEJ than the GM (Fig. 5), despite different tooth groups. Our findings were supported by scatter plots revealing that absolute curvature values of approximately 1 mm^− 1^ represent an arbitrary threshold where higher deviations in measurement point position can be observed (Fig. 6). To outline the magnitude of the problem, half of the cases exhibit CEJ with curvature less than 1 mm^− 1^. Furthermore, a great example is comparing the first and second round of measurements for Examiner No. 1 with larger deviations present at higher absolute curvature values as well in the first round. In contrast, in the second round, a “learning effect” was observed with improving precision over the higher range, but importantly, not below the mentioned threshold.

This study was subject to some limitations. First, either measurement point’s true position is impossible to determine; therefore, the trueness of measurements could not be evaluated. Despite the perfect reproducibility of point selection presented automated approach requires initial human input to determine the points of interest. In the present study, the point definition was based on definitions from existing literature [11, 28, 31–34]. However, with time, both the cement and enamel wears away due to the exposure of tooth’s root surface to the oral environment [17], rendering CEJ a questionable landmark. The automated curvature-based approach is applicable beyond the limitations of identifiable CEJ, meaning that when defects or restorations are present, the edges of the defect or restorations can be objectively defined by curvature analysis as well. Thus, in digital analysis, the term and landmark of CEJ might have been redefined to “coronal reference point” in the scope of the periodontal measurements. Additionally, curvature analysis can also aid in describing root surface defects’ morphology, opening novel insights into the reconstruction of anatomical CEJ before root coverage [37, 38]. However, further research is required for validation in cases without identifiable natural CEJ.

Second, compared to a whole 3D model, a cross-section represents only one out of many available measuring sites. Further research is required to explore novel possibilities analysing whole 3D models. Nonetheless, cross-sections are widely used to evaluate tissue dynamics in periodontal plastic surgery [35, 39–43] and implantology [44–46], enabling great standardisation regarding the selection of measuring site and direction of measurement. Measurement site selection, e.g., selecting a representative cross-section, is also one of the possible variabilities in the measurement of recession depth. For follow-up measurements measuring site variability was eliminated with superimposition of digital models [11, 40], also stating the importance of superimposition accuracy [47].

Third, while the clinical approach utilizes also the colour properties as the additional visual reference, only shape properties were used in the present study. Despite color acquisition of digital dental models in the study, export of color models for further analysis was unavailable due to software limitations. Exporting of color models has become available only recently for some systems and color models proved to be useful in digital measurements of keratinized tissue width with main emphasis on color difference [48]. Further research could be aimed to test the suitability of colour properties on automated CEJ detection.

In periodontology, the potential of digital measurements remains unexploited due to the unclear definitions and inconsistent implementations, inherited from the conventional methods [29]. In treatment planning, the automated digital method would allow for the early detection of initial, i.e., prodromal, recessions due to higher resolution and precision, empowering the appropriate preventive measures. Importantly, the same level of resolution and precision will be maintained through the follow-up period. Such precision through all phases of evaluation will greatly improve the quality as well as the credibility of clinical and research data [29].

## Conclusions

Novel automated approach increases the precision of gingival recession measurements by using curvature analysis, opening novel possibilities for comparison of different treatment techniques. The utilisation of curvature analysis enables completely reproducible measurement point selection, eliminating human variability and seems promising for the evaluation of teeth with indistinguishable CEJ that were often excluded in the previous studies. Therefore, future studies could be done on larger, more clinically relevant samples.

## Supplementary Information

**Additional file 1.** Digital models used in the present study in a STL file format.

**Additional file 2.** Raw measurements and data used in the present study.

**Additional file 3: Figure S1**. Bland-Altman plots showing a comparison of clinical and digital manual measurements of gingival recession between Examiner No. 1 (red dots), No. 2 (blue dots), and No. 3 (green dots) in the first (a, c, and e) and second round of measurements (b, d, and f) in reference to clinical approach. A comparison of clinical and digital automated measurements in reference to clinical approach (g). The x-axis indicates the mean measurement of the gingival recession between compared approaches. The y-axis indicates the difference between compared approaches. A black line with the surrounding grey area indicates mean bias and 95% confidence interval. A dashed black line with a surrounding grey area indicates either upper or lower 95% limits of agreement and corresponding 95% confidence interval.

## Data Availability

Digital models used in the present study are available in this article's Additional file 1. Please cite this article when using the digital models. Additionally, the data supporting the study’s findings, i.e., raw measurements and data, are available in Additional file 2. Bland-Altman plots evaluating agreement of digital and clinical recession depth measurements are available as Additional file 3.

## Abbreviations

3D: Three-dimensional
CEJ: Cemento-enamel junction
GM: Gingival margin.
ICC: Intraclass correlation coefficient.
