# Supplementary material for: The precision of gingival recession measurements is increased by an automated curvature analysis method
Source: BMC Oral Health. 2021 Oct 7;21:505. doi: 10.1186/s12903-021-01858-9 (PMC8499415; doi:10.1186/s12903-021-01858-9)
Supplement: Supplementary file 3 — Additional file 3: Figure S1. Bland-Altman plots showing a comparison of clinical and digital manual measurements of gingival recession between Examiner No. 1 (red dots), No. 2 (blue dots), and No. 3 (green dots) in the first (a, c, and e) and second round of measurements (b, d, and f) in reference to clinical approach. A comparison of clinical and digital automated measurements in reference to clinical approach (g). The x-axis indicates the mean measurement of the gingival recession between compared approaches. The y-axis indicates the difference between compared approaches. A black line with the surrounding grey area indicates mean bias and 95% confidence interval. A dashed black line with a surrounding grey area indicates either upper or lower 95% limits of agreement and corresponding 95% confidence interval. [file 12903_2021_1858_MOESM3_ESM.docx]

**Supplementary material**


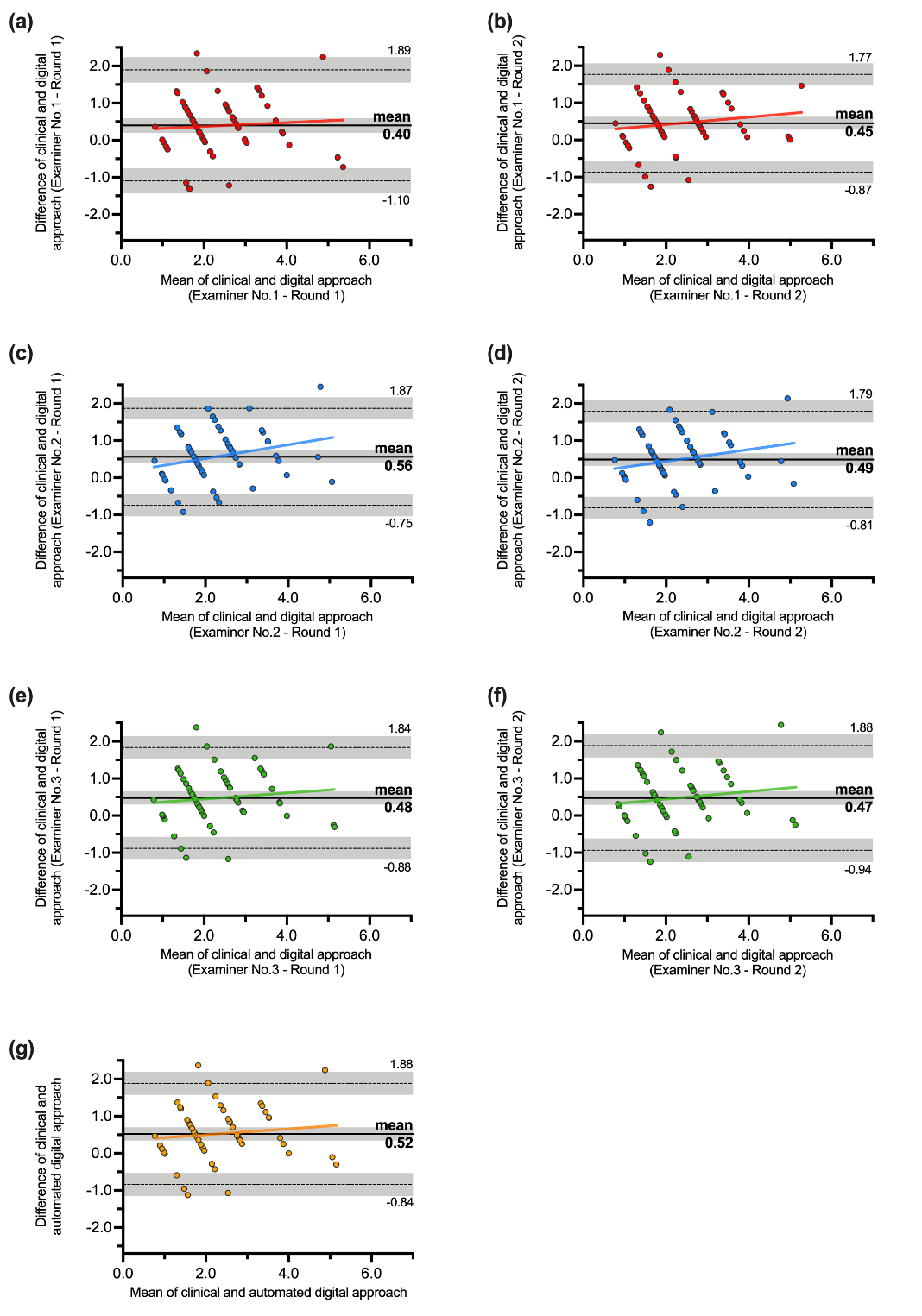


**Supplementary Figure 1:** Bland-Altman plots showing a comparison of clinical and digital manual measurements of gingival recession between Examiner No. 1 (red dots), No. 2 (blue dots), and No. 3 (green dots) in the first (a, c, and e) and second round of measurements (b, d, and f) in reference to clinical approach. A comparison of clinical and digital automated measurements in reference to clinical approach (g). The x-axis indicates the mean measurement of the gingival recession between compared approaches. The y-axis indicates the difference between compared approaches. A black line with the surrounding grey area indicates mean bias and 95% confidence interval. A dashed black line with a surrounding grey area indicates either upper or lower 95% limits of agreement and corresponding 95% confidence interval.
