# Supplementary material for: The precision of gingival recession measurements is increased by an automated curvature analysis method
Source: BMC Oral Health. 2021 Oct 7;21:505. doi: 10.1186/s12903-021-01858-9 (PMC8499415; doi:10.1186/s12903-021-01858-9)
Supplement: Supplementary file 1 — Additional file 1. Digital models used in the present study in a STL file format. [file 12903_2021_1858_MOESM1_ESM.zip › Digital_models/File_legend.doc]

| **Filename (*.stl)** | **Patient** (Number) **& Teeth** (FDI notation) |
| --- | --- |
| **01** | Patient #01 – 16, 15, 14, and 13 |
| **02** | Patient #01 – 23, 24, 25, and 26 |
| **03** | Patient #01 – 34, 33, 41, and 44 |
| **04** | Patient #02 – 14 |
| **05** | Patient #02 – 24 |
| **06** | Patient #02 – 31 and 41 |
| **07** | Patient #03 – 16 and 14 |
| **08** | Patient #03 – 22, 23, 24, and 26 |
| **09** | Patient #04 – 17, 16, 15, and 14 |
| **10** | Patient #05 – 17, 16, 14, 13, and 12 |
| **11** | Patient #05 – 25 |
| **12** | Patient #06 – 16, 15, and 13 |
| **13** | Patient #06 – 26 |
| **14** | Patient #07 – 16, 13, 21, 23, 24, and 25 |
| **15** | Patient #08 – 11 and 21 |
| **16** | Patient #09 – 16 and 14 |
| **17** | Patient #09 – 23 |
| **18** | Patient #10 – 13, 12, and 21 |
| **19** | Patient #10 – 24 |
| **20** | Patient #10 – 25 |
